# Effects of Colony Breeding System and Nest Architecture on Soil Microbiome and Fertility in the Fungus-Growing Termite *Macrotermes barneyi* Light

**DOI:** 10.3390/insects16050470

**Published:** 2025-04-29

**Authors:** Jiachang Zhou, Wenquan Qin, Yang Zeng, Xin Huang, Jing Yuan, Yuting Yin, Paike Xu, Xiaohong Fan, Runfeng Zhang, Ganghua Li, Yinqi Zhang

**Affiliations:** 1 Hubei Key Laboratory of Edible Wild Plants Conservation and Utilization, Hubei Normal University, Huangshi 435002, China; zhoujiachang@stu.hbnu.edu.cn (J.Z.); qinwenquan@hbnu.edu.cn (W.Q.); zengyang73@hbnu.edu.cn (Y.Z.); huangxin@stu.hbnu.edu.cn (X.H.); 2023z08601205@stu.hbnu.edu.cn (J.Y.); yinyuting@stu.hbnu.edu.cn (Y.Y.); xupaike@stu.hbnu.edu.cn (P.X.); fan@stu.hbnu.edu.cn (X.F.); zrfeng163@hbnu.edu.cn (R.Z.); 2 Key Laboratory of Termite Control of Ministry of Water Resources, China Institute of Water Resources and Hydropower Research, Beijing 100048, China

**Keywords:** polygynous, fungus-growing termite, soil fertility, metagenomics, *Macrotermes barneyi*

## Abstract

*Macrotermes barneyi* is a typical fungus-growing termite that forms both monogynous (single queen) and polygynous (multiple queen) colonies in nature. This species influences the local soil fertility in part by redistributing nutrients across the landscape in its habitats. However, how the colony structure of *M. barneyi* affects nutrient cycling and microbial communities within the nest is not well understood. In this study, we compared the physicochemical properties and microbial communities across nest parts between monogynous and polygynous colonies of *M. barneyi*. Our results showed that the fungus garden is the most nutrient-rich part of the nest, with higher soil moisture, organic matter, ammonium nitrogen, nitrate nitrogen, available sulfur, available potassium, available silicon, and available boron than other nest parts. Notably, the fungus garden in monogynous colonies had higher nitrate nitrogen, available sulfur, and available silicon than those in the polygynous colonies. The microbial α-diversity in the fungus garden was lower than that in other parts of the nest. β-diversity analysis revealed a clear separation of microbial communities between monogynous and polygynous colonies across nest parts. Furthermore, the relative abundance of functional genes associated with “cell cycle control, cell division, and chromosome partitioning” was higher in the fungus garden of polygynous colonies compared to monogynous colonies. Our results suggest that the fungus garden plays a crucial role in maintaining colony stability in *M. barneyi* colonies. The rapid depletion of nutrients in the fungus garden to sustain the larger population in polygynous colonies likely influences microbial community dynamics and nutrient cycling.

## Simple Summary

*Macrotermes barneyi* is a fungus-growing termite that forms colonies with either a single queen (monogyne social form) or multiple queens (polygyne social form). These termites redistribute nutrients and shape soil properties through their nesting and foraging activities. This study examined how colony structure influences soil nutrients and microbial communities within the nest. The fungus garden—the area where termites cultivate their symbiotic fungus—was the most nutrient-rich part of the nest, containing higher levels of moisture, organic matter, and key nutrients such as nitrogen, sulfur, potassium, silicon, and boron. Fungus gardens in monogynous colonies had higher concentrations of certain nutrients than those in polygynous colonies. Microbial diversity and community composition also varied between colony types, with some microbial functions more active in polygynous colonies. These findings show that the fungus garden plays a key role in the nest and suggest that differences in colony structure may affect nutrient cycling and microbial dynamics.

## 1. Introduction

Termites are among the most destructive pests globally, causing annual economic losses estimated at up to USD 40 billion [1,2]. Their nest construction, colony expansion, and foraging activities pose significant threats to agricultural and forestry resources and compromise the structural integrity of buildings, including houses and dams [3,4,5]. While termites are widely recognized for their destructive impact, they also play crucial roles in soil regulation and ecosystem health [6]. Through their nest-building and foraging activities, termites regulate soil properties by enhancing structure, increasing porosity, and promoting nutrient cycling [7]. Thus, termites are regarded as soil engineers in various ecosystems, including forests, savannas, wetlands, and even deserts [8,9,10,11,12].

Termites construct diverse nest types, ranging from simple arboreal and subterranean nests to large, intricate cathedral mounds, reflecting their social organization, lifestyles, and ecological adaptations across species [13]. For example, fungus-growing termites cultivate symbiotic fungi in so-called fungus gardens within their mounds or subterranean nests. These nests not only provide shelter for the colony but also significantly influence soil nutrient distribution, hydrological properties, microbial communities, plant diversity, and vegetation growth [6,7,9]. Furthermore, by consuming litter and soil organic matter, termites promote biomass mineralization and soil humification, leading to higher concentrations of macro- and micronutrients in their nests and the surrounding soils compared to unaffected areas. Although extensive research has focused on termite mounds, the structure and ecological functions of subterranean nests remain poorly understood.

Termites are eusocial insects with highly organized colonies. Unlike ants and bees, termite societies are mixed-sex. A termite colony is typically founded by a monogynous pair of alates (monogyny) during swarming. However, multiple queens in a single colony (polygyny) were also found in many termite species, especially in lower termite societies [14]. Generally, polygynous colonies always have higher genetic diversity and larger populations than monogynous colonies [15,16]. These attributes enhance the resilience of polygynous colonies in response to environmental challenges and accelerate their expansion in nature. However, polygynous associations may also cause conflicts among queens, although cooperation can provide significant advantages during the early stage of colony establishment. For example, in the termite *Cornitermes cumulans*, polygynous colonies have a higher number of eggs but fewer larvae, along with greater difficulty in maintaining stability during the early development of colonies compared to monogynous colonies [17]. Polygyny is an important breeding phenomenon in eusocial insects. Understanding this phenomenon provides insights into the social organization, collective behavior, and genetic diversity of insect societies. However, our knowledge of polygynous colonies in termites is limited.

**Figure 1:**
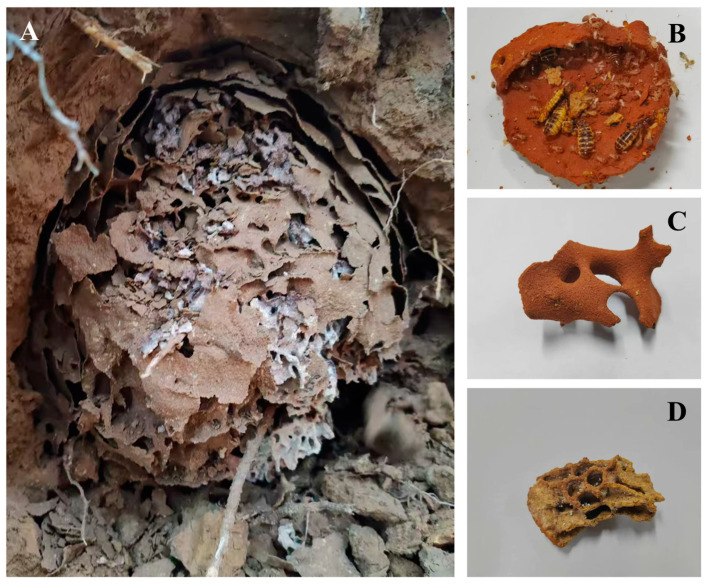
Photographs showing different parts of a *Macrotermes barneyi* nest, including the excavated nest (**A**), the royal chamber (**B**), the soil skeleton (**C**), and the fungus garden (**D**).

This study focuses on the fungus-growing termite *Macrotermes barneyi* Light, a significant pest that poses a serious threat to dam safety in China [18,19,20]. This termite has both monogyny and polygyny breeding strategies in nature [21]. Their subterranean nest comprises three distinct biostructures: the fungus garden, used for cultivating symbiotic fungi; the soil skeleton, which provides structural support; and the royal chamber, where the king and queen reside (Figure 1). Notably, there is no obvious morphological difference in the organization of these three structures between monogynous and polygynous colonies. As ecosystem engineers, fungus-growing termites (Macrotermitinae) significantly alter their environment by locally enriching soil nutrients and modifying soil texture and moisture [6,22,23], thereby creating spatial heterogeneity that can influence microbial communities [24]. However, there is limited information on how fungus-growing termites influence soil microbes within their subterranean nests, particularly in relation to their colony breeding system and internal nest architecture.

In this study, we compare the physicochemical properties and microbial communities of the fungus garden, soil skeleton, royal chamber, and ambient soil between monogynous and polygynous colonies of *M. barneyi*. We hypothesize that polygynous colonies, due to their larger population and complex social structure, will exhibit distinct physicochemical characterizations and microbial communities compared to monogynous colonies. Our findings provide insight into the adaptive strategies of polygynous colonies and contribute to the development of effective and ecological management strategies for *M. barneyi*.

## 2. Materials and Methods

### 2.1. Soil Sampling

Three monogynous and three polygynous colonies of *M. barneyi* were collected in May 2021 from a chestnut forest in Linchuan District of Fuzhou City in Jiangxi Province, central China. After excavating the main colony, the royal chamber was carefully removed. A scalpel was used to gently make a three-quarter incision along the side central line of the royal chamber, and the upper section was carefully pried open. This semi-open, non-destructive opening allowed for accurate counting of breeding females with minimal visual obstruction while maintaining the structural integrity of the chamber for transport. The number of breeding females was counted and recorded to determine the colony type (Figure 1).

After the colony type was confirmed, the soil samples were placed individually in self-sealing bags, which were then stored in an ice box with ice packs for short-term preservation before being transported back to the laboratory within two days. Upon arrival, all samples were processed immediately to minimize any potential changes. Samples from the royal chamber, soil skeleton, fungus garden, and ambient soil (5 m from the main colony) were collected using the five-point sampling method. For each sample type, five evenly distributed sampling points were selected around the center, and approximately 20 g of material was excavated from each point, yielding a total of ~100 g per colony. This ensured uniformity and minimized external influences. Furthermore, the lower surface adjacent to the royal chamber was used as the reference horizontal plane. Each colony was sampled only once to avoid disturbance and ensure independent replicates.

### 2.2. Soil Physicochemical Analysis

The soil pH was measured using a 1:2.5 soil-to-water ratio (*w*/*v*) with a glass electrode pH meter. Soil water content was determined by drying samples for 72 h at 105 °C. The contents of soil organic matter (OM), ammonium nitrogen (N), nitrate nitrogen (N), available sulfur (S), available potassium (K), available silicon (Si), and available boron (B) were measured using a spectrophotometric method with the corresponding kits (Lai Er Bio-Tech Co., Ltd., Hefei, China).

### 2.3. Metagenome DNA Extraction and Shotgun Sequencing

Total microbial genomic DNA samples were extracted from 0.3 g of each sample using the Soil DNA Kit (Omega Bio-tek, Inc., Norcross, GA, USA) following the manufacturer’s instructions and stored at −20 °C until further assessment. The quantity and quality of extracted DNA were measured using a NanoDrop ND-1000 spectrophotometer (Thermo Fisher Scientific, Waltham, MA, USA) and agarose gel electrophoresis, respectively. The extracted microbial DNA was processed to construct metagenome shotgun sequencing libraries with insert sizes of 400 bp by using the Illumina TruSeq Nano DNA LT Library Preparation Kit. Each library was sequenced using the Illumina HiSeq X-ten platform (Illumina, San Diego, CA, USA) with PE150 strategy at Personal Biotechnology Co., Ltd. (Shanghai, China).

### 2.4. Metagenomics Analysis

Raw sequencing reads were processed to obtain quality-filtered reads for further analysis. First, sequencing adapters were removed from sequencing reads using Cutadapt (v1.2.1) [25]. Secondly, low-quality reads were trimmed using a sliding-window algorithm in fastp [26]. Once quality-filtered reads were obtained, taxonomical classifications of metagenomics sequencing reads from each sample were performed using Kraken2 against a RefSeq-derived database, which included genomes from archaea, bacteria, viruses, fungi, protozoans, metazoans, and viridiplantae [27]. Reads assigned to metazoans or viridiplantae were removed for downstream analysis [28]. Megahit (v1.1.2) was used to assemble each sample using the meta-large preset parameters [29]. The generated contigs (longer than 200 bp) were then pooled together and clustered using mmseqs2 with “easy-linclust” mode, setting the sequence identity threshold to 0.95 and covering the residues of the shorter contig by 90% [30]. The lowest common ancestor taxonomy of the non-redundant contigs was obtained by aligning them against the NCBI-nt database by mmseqs2 with “taxonomy” mode, and contigs assigned to Viridiplantae or Metazoa were dropped in the following analysis [30]. MetaGeneMark was used to predict the genes in the contigs [31]. CDS sequences of all samples were clustered by mmseqs2 with “easy-cluster” mode, setting the protein sequence identity threshold to 0.90 and covering the residues of the shorter contig by 90% [30]. To assess the abundance of these genes, the high-quality reads from each sample were mapped onto the predicted gene sequences using salmon in the quasi-mapping-based mode with “-meta –minScoreFraction = 0.55”, and the CPM (copy per kilobase per million mapped reads) was used to normalize abundance values in metagenomes [32]. The functionality of the non-redundant genes was obtained by annotation using mmseqs2 with the “search” mode against the protein databases of KEGG, EggNOG, and CAZy, respectively [28]. EggNOG and GO were obtained using EggNOG-mapper (v2) [33]. GO ontology was obtained using map2slim (www.metacpan.org) (accessed on 3 November 2021). KO was obtained using KOBAS (V3.0) [34].

### 2.5. Statistical Analysis

Based on the taxonomic and functional profiles of non-redundant genes, LEfSe (linear discriminant analysis effect size) was performed to detect differentially abundant taxa and functions across groups using the default parameters, where the term “biomarker” refers to features with significantly different relative abundances between groups [35]. The Shapiro–Wilk test was used to detect whether the data exhibited normal distribution. Transformed data were used for analysis if the raw data did not confirm the normal distribution. The soil physicochemical properties were compared using two-way ANOVA followed by Tukey’s HSD test for multiple comparison. The significance was determined at α < 0.05. All statistical analyses were conducted using IBM SPSS Statistical 24.0 (SPSS Inc., Chicago, IL, USA).

## 3. Results

### 3.1. Soil Physicochemical Characterization

**Figure 2:**
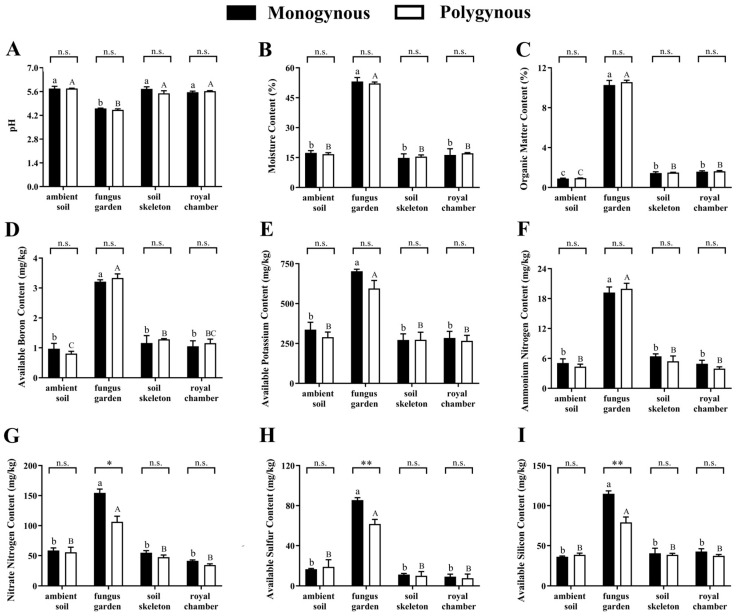
Soil physicochemical properties (mean ± SE, *n* = 3) across nest parts in both monogynous and polygynous colonies of *Macrotermes barneyi*. (**A**) pH, (**B**) moisture content, (**C**) organic matter content, (**D**) available boron content, (**E**) available potassium content, (**F**) ammonium nitrogen content, (**G**) nitrate nitrogen content, (**H**) available sulfur content, (**I**) available silicon content. Different lowercase and uppercase letters on the tops of columns indicate significant differences among nest parts in monogynous and polygynous colonies, respectively (*p* < 0.05). Asterisks represent significant differences (* *p* < 0.05, ** *p* < 0.01); n.s. represents no significant difference.

Soil pH was not significantly affected by the breeding system (*F* = 1.887, *p* = 0.188) or by its interaction with the nest part (*F* = 2.026, *p* = 0.151), but varied significantly across nest parts (*F* = 119.501, *p* < 0.001). The soil pH of the fungus garden was significantly lower than other nest parts (Figure 1A). Soil moisture (*F* = 0.002, *p* = 0.967), soil OM (*F* = 1.375, *p* = 0.258), available B (*F* = 0.202, *p* = 0.659), available K (*F* = 3.772, *p* = 0.070), and ammonium N content (*F* = 0.690, *p* = 0.418) were not significantly affected by the breeding system or by its interaction with the nest part (*F* = 0.411, *p* = 0.747; *F* = 0.482, *p* = 0.699; *F* = 0.456, *p* = 0.717; *F* = 1.156, *p* = 0.357; *F* = 0.522, *p* = 0.673, respectively), but varied significantly across nest parts (*F* = 657.155, *p* < 0.001; *F* = 2671.706, *p* < 0.001; *F* = 113.143, *p* < 0.001; *F* = 67.293, *p* < 0.001; *F* = 158.195, *p* < 0.001, respectively). The contents of moisture, OM, available B, available K, and ammonium N in the fungus garden were significantly higher than those in other nest parts (Figure 2B–F). Soil nitrate N (*F* = 7.400, *p* = 0.003), available S (*F* = 11.309, *p* < 0.001), and available Si (*F* = 14.388, *p* < 0.001) contents were significantly affected by the interaction between the breeding system and the nest parts. The contents of nitrate N (*t* = 4.260, *p* = 0.013), available S (*t* = 6.555, *p* = 0.003), and available Si (*t* = 6.803, *p* = 0.002) in the fungus gardens of monogynous colonies were significantly higher than those in the polygynous colonies. However, no significant effect was found in the other nest parts (Figure 2G–I).

### 3.2. Microbial Community Composition

**Figure 3:**
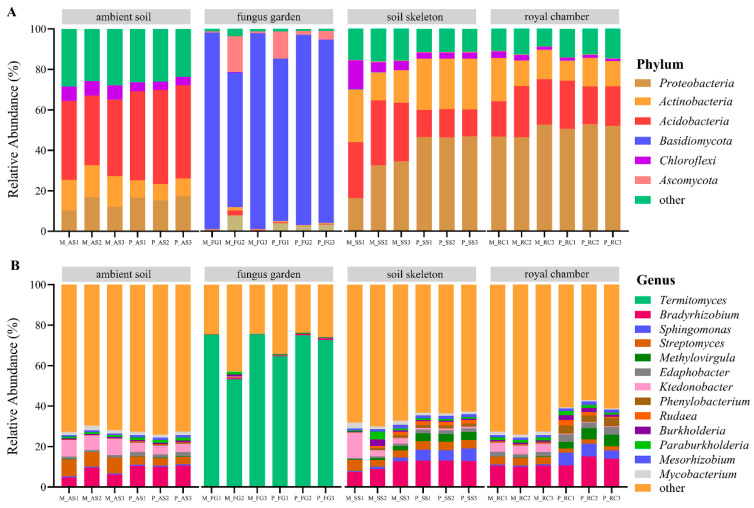
Microbial community composition across nest parts in both monogynous and polygynous colonies of *Macrotermes barneyi*. The relative abundance of the (**A**) top 20 phyla; (**B**) top 20 genera. Abbreviations used: M, monogynous colony; P, polygynous colony; AS, ambient soil; FG, fungus garden; SS, soil skeleton; RC, royal chamber.

At the kingdom level, all soil samples consisted of Bacteria, Eukaryotes, and Archaea. The specific proportions for ambient soil were 98.10% Bacteria, 1.81% Archaea, and 0.09% Eukaryotes; for the fungus garden, they were 94.29% Eukaryotes, 5.70% Bacteria, and 0.01% Archaea; for the soil skeleton, they were 98.92% Bacteria, 0.56% Eukaryotes, and 0.52% Archaea; and for the royal chamber, they were 99.46% Bacteria, 0.38% Eukaryotes, and 0.16% Archaea. A total of 185 phyla and 4249 genera were identified. At the phylum level, there were four phyla with average relative abundances above 10% (Figure 3A), which were Proteobacteria (26.31%), Basidiomycota (21.94%), Acidobacteria (21.15%), and Actinobacteria (12.13%), respectively. At the genus level, there were a total of 13 genera with an average relative abundance above 1% (Figure 3B), which were *Termitomyces* (17.35%), *Bradyrhizobium* (8.32%), *Streptomyces* (3.13%), *Ktedonobacter* (2.53%), *Sphingomonas* (2.15%), *Methylovirgula* (1.88%), *Edaphobacter* (1.46%), *Phenylobacterium* (1.16%), *Paraburkholderia* (1.09%), *Mycobacterium* (1.05%), *Rudaea* (1.04%), *Mesorhizobium* (1.03%), and *Burkholderia* (1.00%), respectively.

The dominant phylum in the fungus garden was Basidiomycota, with a relative abundance of up to 97.17%. At the genus level, the dominant genus in the fungus garden was *Termitomyces*, with a relative abundance of up to 75.40% (Figure 3). Compared to other nest parts, the relative abundances of Basidiomycota and *Termitomyces* in the fungus garden of both monogynous and polygynous colonies were significantly higher (*p* < 0.05). Regardless of whether the colonies were monogynous or polygynous, the relative abundances of Proteobacteria and Acidobacteria in the soil skeleton and royal chamber were significantly higher than that in the fungus garden (*p* < 0.05). At the same time, the relative abundance of *Bradyrhizobium* in the fungus garden of monogynous and polygynous colonies was significantly lower than that in other nest parts (*p* < 0.05; Figure 3).

**Figure 4:**
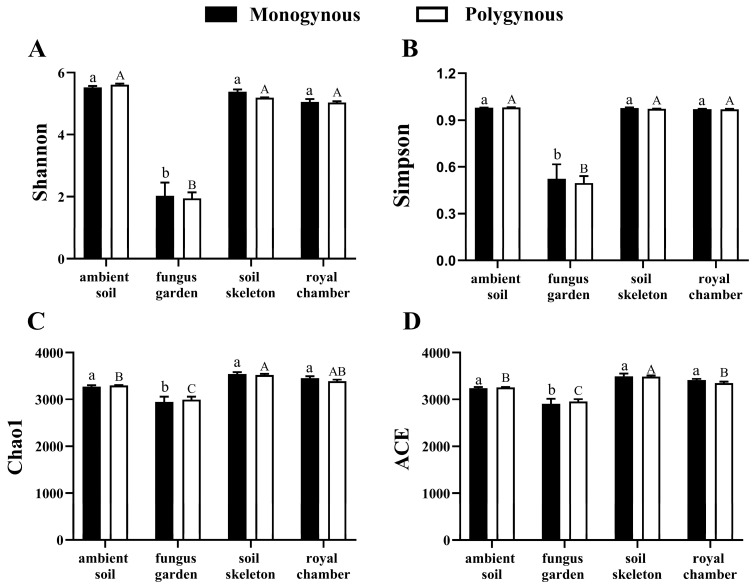
Microbial community diversity across nest parts in both monogynous and polygynous colonies of *Macrotermes barneyi*. Different lowercase and uppercase letters on the tops of columns indicate significant differences among nest parts in monogynous and polygynous colonies, respectively (*p* < 0.05). (**A**) Shannon index; (**B**) Simpson index; (**C**) Chao1 index; (**D**) ACE index.

**Figure 5:**
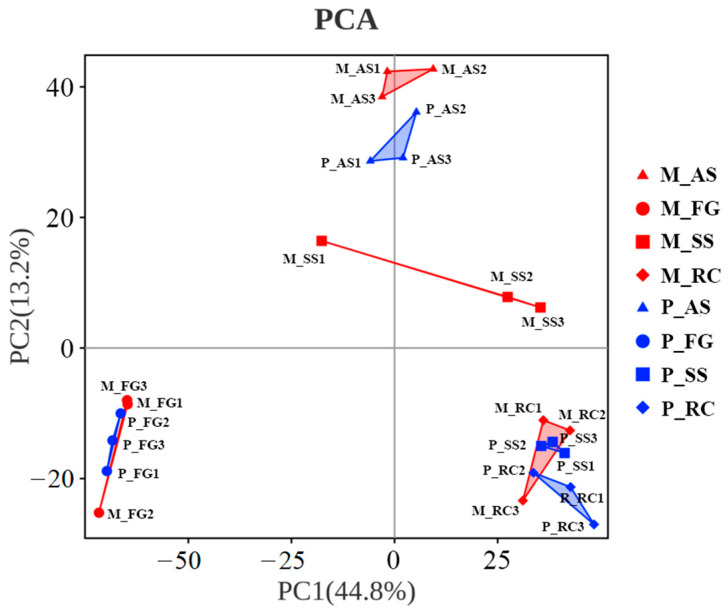
Principal component analysis (PCA) of the soil microbial community across nest parts in both monogynous and polygynous colonies of *Macrotermes barneyi*. Abbreviations used: M, monogynous colony; P, polygynous colony; AS, ambient soil; FG, fungus garden; SS, soil skeleton; RC, royal chamber.

The microbial α-diversity in the fungus garden of both monogynous and polygynous colonies was lower than that in other nest parts (Figure 4). The richness of the microbial community in the colony was not significantly affected by the breeding system (Chao1: *F* = 0.006, *p* = 0.939; ACE: *F* = 0.028, *p* = 0.870) or by its interaction with the nest part (Chao1: *F* = 0.460, *p* = 0.714; ACE: *F* = 0.684, *p* = 0.575), but varied significantly across nest parts (Chao1: *F* = 42.450, *p* < 0.001; ACE: *F* = 39.535, *p* < 0.001). The diversity indices of the microbial community in the colony were not significantly affected by the breeding system (Shannon: *F* = 0.197, *p* = 0.663; Simpson: *F* = 0.082, *p* = 0.779) or by its interaction with the nest part (Shannon: *F* = 0.232, *p* = 0.873; Simpson: *F* = 0.063, *p* = 0.978), but varied significantly across nest parts (Shannon: *F* = 183.689, *p* < 0.001; Simpson: *F* = 80.939, *p* < 0.001). Principal component analysis (PCA) showed that the soil skeleton and ambient soil were distinct from each other between the monogynous and polygynous colonies (Figure 5).

Four biomarkers were identified among the royal chambers of both monogynous and polygynous colonies. Actinobacteria was the dominant biomarker in the royal chamber of monogynous colonies, whereas Caulobacterales was the dominant biomarker in the royal chamber of polygynous colonies (Figure S1). Seventeen biomarkers were identified in the soil skeletons of monogynous and polygynous colonies. The main biomarkers were Chloroflexi and Solibacteres in the soil skeletons of monogynous colonies, whereas Proteobacteria was the main biomarker in the soil skeletons of polygynous colonies (Figure S2). In the ambient soil of the monogynous and polygynous colonies, a total of 19 biomarkers were identified. The dominant biomarkers were Chloroflexi and Actinobacteria in the ambient soil of monogynous colonies, while the dominant biomarkers were Proteobacteria and Acidobacteria in the polygynous colonies (Figure S3). No biomarkers were detected in the fungus garden.

### 3.3. Functional Annotation of eggNOG

**Figure 6:**
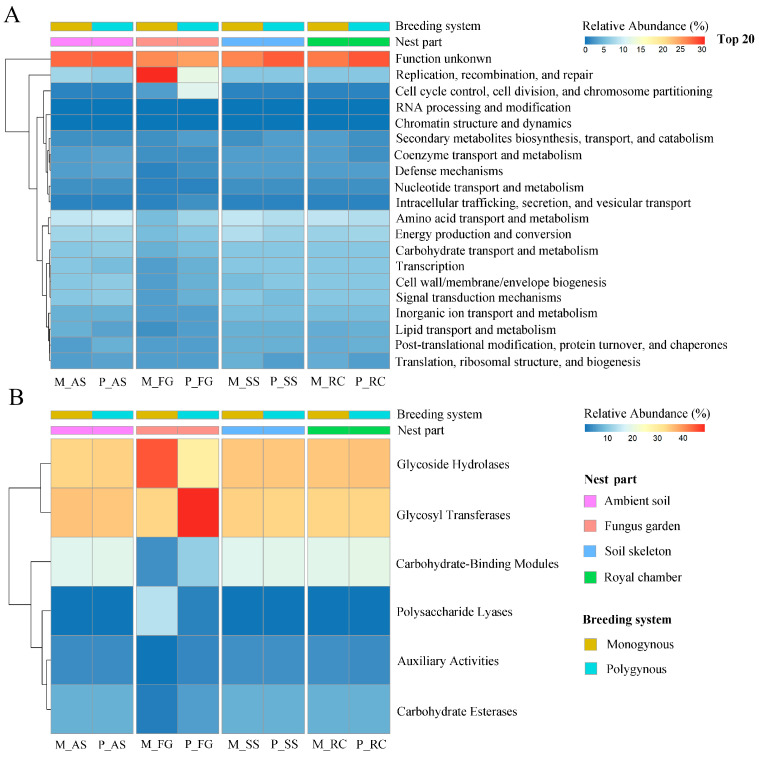
Functional annotation of the soil microbial communities across nest parts in both monogynous and polygynous colonies of *Macrotermes barneyi*. (**A**) Top 20 eggNOG; (**B**) CAZys.

Among all annotated functions, excluding unknown functions, only the pathways “replication, recombination, and repair” and “cell cycle control, cell division, and chromosome partitioning” had a relative abundance exceeding 10% (Figure 6A). The relative abundance of “replication, recombination, and repair” in the colony was not significantly affected by the breeding system (*F* = 2.604, *p* = 0.126) or by its interaction with the nest part (*F* = 2.516, *p* = 0.095), but varied significantly across nest parts (*F* = 5.678, *p* = 0.008). The relative abundance of the “cell cycle control, cell division, and chromosome partitioning” function in the colony was significantly affected by the interaction between the breeding system and nest parts (*F* = 17.717, *p* < 0.001). In polygynous colonies, the relative abundance of the “cell cycle control, cell division, and chromosome partitioning” function in the fungus garden was significantly higher than in the ambient soil, soil skeleton, and royal chamber (*p* < 0.05). However, this function in the fungus garden of monogynous colonies was not different among these nest parts (*p* > 0.05).

### 3.4. Functional Annotation of CAZys

The relative abundance of Auxiliary Activities (AA) (*F* = 0.977, *p* = 0.338), Carbohydrate-Binding Modules (CBM) (*F* = 1.386, *p* = 0.256), Carbohydrate Esterases (CE) (*F* = 1.006, *p* = 0.331), and Polysaccharide Lyases (PL) (*F* = 2.461, *p* = 0.136) were not significantly affected by the breeding system or by its interaction with the nest part (*F* = 1.429, *p* = 0.271; *F* = 0.965, *p* = 0.433; *F* = 1.546, *p* = 0.241; *F* = 2.683, *p* = 0.082, respectively), but varied significantly across nest parts (*F* = 4.415, *p* = 0.019; *F* = 15.950, *p* < 0.001; *F* = 9.466, *p* = 0.001; *F* = 4.564, *p* = 0.017, respectively). The relative abundance of Glycoside Hydrolases (GH) in the colony was significantly affected by the interaction between the breeding system and nest parts (*F* = 10.974, *p* < 0.001). In polygynous colonies, the relative abundance of GH in the fungus garden was significantly lower than in the soil skeleton and royal chamber (*p* < 0.05). However, the relative abundance of GH in the fungus garden of monogynous colonies was significantly higher than in the other nest parts (*p* < 0.05). The relative abundance of Glycosyl Transferases (GT) in the colony was significantly affected by the interaction between the breeding system with the nest parts (*F* = 24.205, *p* < 0.001). In polygynous colonies, the relative abundance of GT in the fungus garden was significantly higher than in the other nest parts (*p* < 0.05). However, the relative abundance of GT in the fungus garden of monogynous colonies was not different among these nest parts (*p* > 0.05; Figure 6B).

### 3.5. Functional Annotation of KEGG

**Figure 7:**
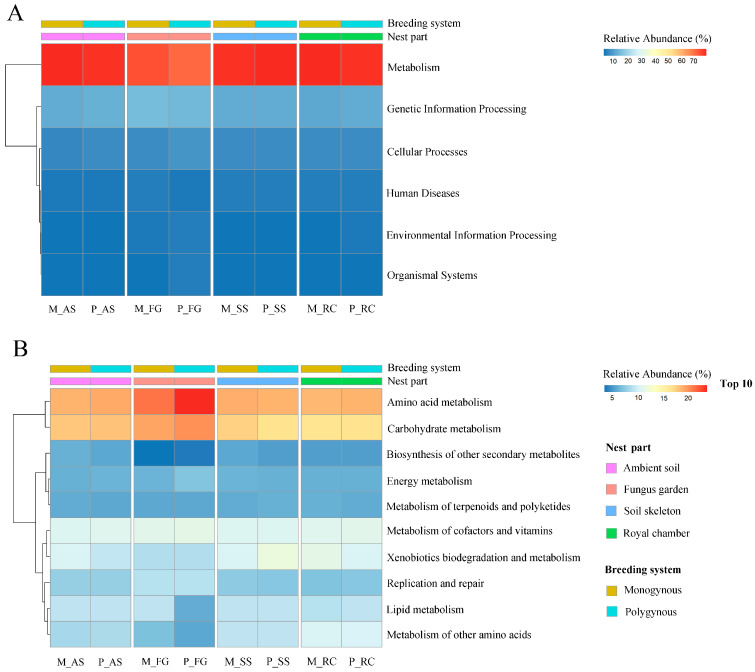
KEGG annotation of the soil microbial communities across nest parts in both monogynous and polygynous colonies of *Macrotermes barneyi*. (**A**) Level 1; (**B**) Level 2.

Among all annotated pathways at KEGG level 1, the pathways “metabolism” and “genetic information processing” (Figure 7A) were the only ones with a relative abundance exceeding 10%. The relative abundance of both “metabolism” and “genetic information processing” in the colony was not significantly affected by the breeding system (*F* = 0.491, *p* = 0.494; *F* = 0.649, *p* = 0.432, respectively) or by its interaction with the nest part (*F* = 0.226, *p* = 0.877; *F* = 0.618, *p* = 0.613, respectively), but varied significantly across nest parts (*F* = 21.176, *p* < 0.001; *F* = 11.129, *p* < 0.001, respectively).

Among all annotated pathways at KEGG level 2, the pathways “amino acid metabolism” and “carbohydrate metabolism” (Figure 7B) were the only ones with a relative abundance exceeding 10%. The relative abundance of “amino acid metabolism” and “carbohydrate metabolism” in the colony was not significantly affected by the breeding system (*F* = 0.019, *p* = 0.892; *F* = 1.067, *p* = 0.317, respectively) or by its interaction with the nest part (*F* = 0.182, *p* = 0.907; *F* = 0.426, *p* = 0.737, respectively), but varied significantly across nest parts (*F* = 7.243, *p* = 0.003; *F* = 8.451, *p* = 0.001, respectively). The relative abundance of “amino acid metabolism” function in the fungus garden was significantly higher than among these nest parts in polygynous colonies (*p* < 0.05). In both monogynous and polygynous colonies, the relative abundance of the “metabolism of other amino acids” function in the fungus garden was lower than in the ambient soil, soil skeleton, and royal chamber (*p* < 0.05).

## 4. Discussion

Termites are key soil ecosystem engineers that influence organic matter and nutrients. In African savannas, fungus-growing termites alter soil nutrient contents through mound construction, resulting in compositionally distinct bacterial and fungal communities in mound topsoils compared to surrounding soils. This activity significantly enhances bacterial diversity, increasing microbial richness and spatial heterogeneity at the landscape scale [24]. In this study, it was also observed that the soil nutrients and moisture in the fungus garden were significantly higher than those in the ambient soil, soil skeletons, and royal chamber, both in monogynous and polygynous colonies. This suggests that *M. barneyi* influences the local nutrient distribution in part by redistributing nutrients across whole subterranean nests. The increased fertility of the fungus garden may enhance the growth of symbiotic fungi, thereby supporting their ability to meet the nutritional needs of the termite colony. The use of termite mound materials as fertilizer is common in Africa, suggesting that fungus gardens of *M. barneyi* or other fungus-growing termites also could serve as valuable sources of nutrients in future soil fertilization efforts [36]. In addition, the *Termitomyces eurrhizus* isolated from the fruiting body of the *T. albuminosus* had an optimal pH of 5.0 during laboratory cultivation [37]. Similarly, the pH of the fungus garden in this study was 4.56. This suggests that under the modification of *M. barneyi*, the fungus garden provides favorable conditions for the growth of symbiotic fungi.

Interestingly, the nutrient fertility in the fungus garden is higher in monogynous colonies than in polygynous colonies. Polygynous colonies are often thought to have higher reproductive capacity due to the multiple queens [15,38]. For example, in *M. michaelseni*, studies have shown that the total number of offspring produced by all the queens in the polygynous colonies is greater than that in the monogynous colonies [17,39]. The fungus garden serves as the “farmland” for the growth of symbiotic fungi by termites, with fungal nodules providing essential nutrients for termites within the colony [40]. Consequently, polygynous colonies require a higher yield from the fungus garden to meet their increased nutritional demands. In conventional agriculture, soil fertility improvements and dense planting can temporarily boost crop yields [41,42]. However, in this study, several fertility-related indicators in the fungus gardens of polygynous colonies were significantly lower than those in monogynous colonies. Given that termite activity enhances soil fertility and that polygynous colonies host larger termite populations, it is speculated that in polygynous colonies, the positive effect of termites on fungus garden fertility may be outweighed by the higher nutrient consumption of symbiotic fungi.

The dominant microbes in the fungus garden are the symbiotic fungi of termites. The composition and diversity of soil microbes are key indicators of soil health and ecological function [43,44]. Generally, the higher the microbial diversity in the soil, the stronger the adaptability to environmental changes [45]. In this study, we found that the microbial diversity in the fungus garden was significantly lower than that of other nest parts. This suggests that the fungus garden may have strong specificity or specialized ecological functions. *Termitomyces* species form a specific mutualistic association with Macrotermitinae termites [46,47]. We thus assume that the main ecological function of the fungus garden in a *M. barneyi* colony is to provide a habitat for the growth of these symbiotic fungi. No biomarkers were found in the fungus garden between the monogynous and polygynous colonies. In contrast, several biomarkers were identified in the ambient soil, soil skeleton, and royal chamber, with a greater number found in the ambient soil and soil skeleton. These results suggest that differences in the colony breeding system may have a stronger impact on soil-associated microbial communities than on those within the fungus garden. The absence of biomarkers in the fungus garden might be related to its relatively stable microbial composition or to limitations in sample size and sampling resolution. Future studies could increase the sample size and expand sampling locations to further address these limitations and provide more comprehensive insights.

The fungus garden in polygynous colonies may meet the colony’s food demands through rapid proliferation. Functions related to “cell cycle control, cell division, chromosome partitioning” are closely associated with cellular proliferation [48]. We found that the relative abundance of these functions in the polygynous colony was significantly higher than that in the monogynous colony. These results suggest that intense microbial proliferation activities may occur in polygynous colonies. Future research could further explore the role of these functions in maintaining balance within polygynous colonies by comparing the growth rates and densities of the symbiotic fungi.

Carbohydrate-Active Enzymes (CAZymes) are closely linked to their saprophytic lifestyle in fungi [49,50]. In this study, we observed that GT and GH, two types of CAZymes, were highly abundant across all nest parts. However, the concentration of GT in polygynous colonies was significantly higher than that in monogynous colonies, while the GH concentration was lower in the fungus garden of polygynous colonies compared to those of monogynous colonies. These findings indicate that there are differences in carbohydrate metabolism in the fungus garden between monogynous and polygynous colonies. Comparing these functional differences across samples helps to understand the roles, interactions, and adaptive mechanisms within their ecosystem [51,52]. Additionally, this study revealed that the “metabolism of other amino acids” function in the fungus gardens of both monogynous and polygynous colonies was significantly lower than in the ambient soil, soil skeleton, and royal chamber. However, in polygynous colonies, “amino acid metabolism” in the fungus garden was significantly higher than in other nest parts. These results suggest that amino acid metabolism may play a crucial role in maintaining the balance of the *M. barneyi* colonies.

In summary, our analysis of soil nutrients, microbial communities, and functional metagenomics revealed that the fungus garden plays a crucial role in maintaining the ecological balance of *M. barneyi* colonies. We found that polygynous colonies adapt to greater nutrient demands through enhanced microbial proliferation and distinct metabolic adjustments. Compared to monogynous colonies, nutrient depletion in the fungus gardens of polygynous colonies may occur more rapidly. These findings not only deepen our understanding of termite–fungus symbiosis but also provide valuable insights into the role of fungus gardens in pest control strategies, offering potential applications for sustainable agriculture and soil fertility improvement.

## Data Availability

The data that support the findings of this study are available from the corresponding author upon reasonable request. Furthermore, the sequence data reported in this paper have been deposited in the NCBI SRA database (Bio Project ID: PRJNA1218466).

## Supplementary Materials

The following supporting information can be downloaded at: https://www.mdpi.com/article/10.3390/insects16050470/s1, Figure S1: Linear discriminant analysis effect size (LEfSe) results for microbial communities associated with the royal chambers of *Macrotermes barneyi* nests, comparing monogynous and polygynous colonies (LDA score threshold = 4.0). (A) Cladogram of discriminative taxa; (B) Histogram of LDA scores; Figure S2: Linear discriminant analysis effect size (LEfSe) results for microbial communities associated with the soil skeletons of *Macrotermes barneyi* nests between monogynous and polygynous colonies (LDA score threshold = 4.0). (A) Cladogram of discriminative taxa; (B) Histogram of LDA scores; Figure S3: Linear discriminant analysis effect size (LEfSe) results for microbial communities associated with the ambient soils of *Macrotermes barneyi* nests, comparing monogynous and polygynous colonies (LDA score threshold = 4.0). (A) Cladogram of discriminative taxa; (B) Histogram of LDA scores.
