# Supplementary material for: Effects of Colony Breeding System and Nest Architecture on Soil Microbiome and Fertility in the Fungus-Growing Termite Macrotermes barneyi Light
Source: Insects. 2025 Apr 29;16(5):470. doi: 10.3390/insects16050470 (PMC12111960; doi:10.3390/insects16050470)
Supplement: Supplementary file 1 [file insects-16-00470-s001.zip › insects-3596159-supplementary.pdf]

Supplementary Material

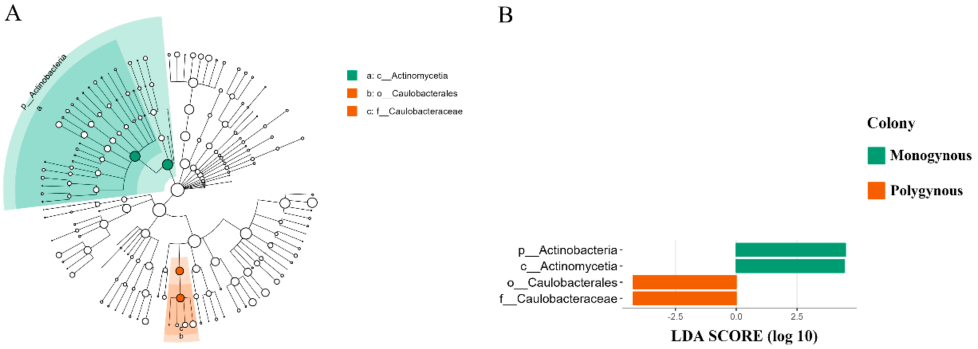

Figure S1. Linear discriminant analysis effect size (LEfSe) results for microbial communities associated with the royal chambers of *Macrotermes barneyi* nests, comparing monogynous and polygynous colonies (LDA score threshold = 4.0). (A) Cladogram of discriminative taxa; (B) Histogram of LDA scores.

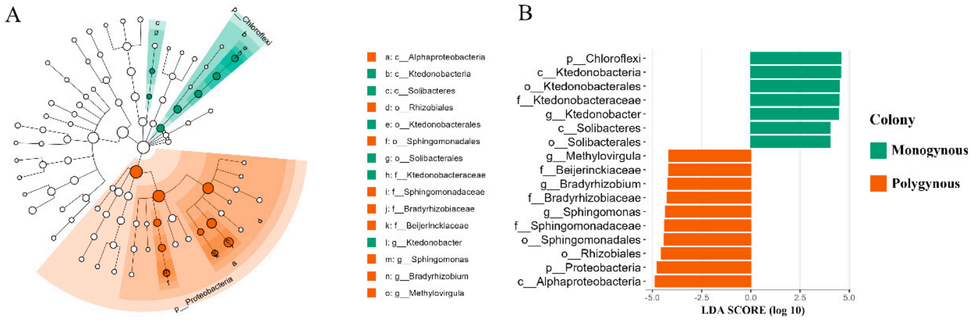

Figure S2. Linear discriminant analysis effect size (LEfSe) results for microbial communities associated with the soil skeletons of *Macrotermes barneyi* nests, comparing monogynous and polygynous colonies (LDA score threshold = 4.0). (A) Cladogram of discriminative taxa; (B) Histogram of LDA scores.

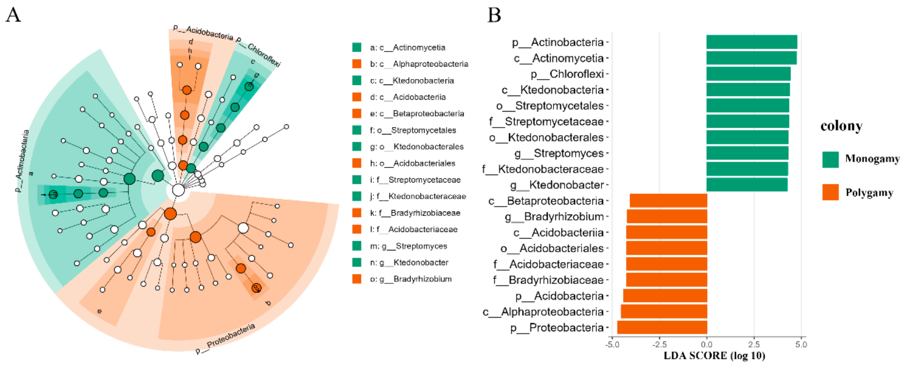

Figure S3. Linear discriminant analysis effect size (LEfSe) results for microbial communities associated with the ambient soils of *Macrotermes barneyi* nests, comparing monogynous and polygynous colonies (LDA score threshold = 4.0). (A) Cladogram of discriminative taxa; (B) Histogram of LDA scores.
